# The asymmetrical force of persuasive knowledge across the positive–negative divide

**DOI:** 10.3389/fpsyg.2015.01324

**Published:** 2015-09-04

**Authors:** Mads Nordmo, Marcus Selart

**Affiliations:** Department of Strategy and Management, The Norwegian School of EconomicsBergen, Norway

**Keywords:** persuasion, resistance, CSR, negativity bias, persuasion knowledge, attribute framing, numerosity, dilution effect

## Abstract

In two experimental studies we explore to what extent the general effects of positive and negative framing also apply to positive and negative persuasion. Our results reveal that negative persuasion induces substantially higher levels of skepticism and awareness of being subjected to a persuasion attempt. Furthermore, we demonstrate that in positive persuasion, more claims lead to stronger persuasion, while in negative persuasion, the numerosity of claims carries no significant effect. We interpret this finding along the lines of a satiety-model of persuasion. Finally, using diluted, or low strength claims in a persuasion attempt, we reveal a significant interaction between dispositional reactance and dilution of claims on persuasion knowledge. The interaction states that diluted claims increase the awareness of being subjected to a persuasion attempt, but only for those with a high dispositional level of reactance.

## Introduction

The purpose of this research is threefold: Firstly, it demonstrates that resistance and skepticism to persuasion is not symmetrical across the positive–negative divide. Secondly, it demonstrates that the number of claims have different effects in positive and negative persuasion. Thirdly, it brings about important managerial implications for corporate social responsibility (CSR) communication in particular, and persuasion in general. The main research-question addressed by this paper is: What are the effects of different numbers of claims in positive and negative persuasion? The second, related research-question is: how do different qualities of claims affect the outcomes in positive and negative persuasion? These questions are explored in two experiments, using CSR communications as stimuli.

In CSR research in particular, and in psychology in general, there is a heightened need to better understand the evaluative artifacts of positive and negative persuasion. Corporations today are commonly considered to have social responsibility to serve the people, communities, society, and the environment in ways that go above and beyond what is legally required (Wood, 1991; McWilliams and Siegel, 2001; Lockett et al., 2006). As a consequence, corporations now face the opportunity to communicate all their socially and environmentally laudable efforts in order to increase their likeability. However, they also face the risk of negative attention reaching their less prize-worthy activities. Individuals and organizations thus face a perilous and delicate situation. By under-communicating CSR activities, one faces the risk of people never learning about the activities, and possibly assuming that no CSR initiatives have been made. By over-communicating CSR activities, one faces the risk of skepticism and cynicism on behalf of weary consumers, who disbelieve the accuracy and sincerity of the claims. There is a far-reaching string of literature regarding how the number of claims, or arguments, affect the outcome of persuasion. The idea that additional positive information increases liking has been largely supported in psychological research on attribution and impression formation. Stewart (1965) found that the when participants are presented with a description of a person consisting of one, two, three or four positive traits, and subsequently four negative traits, the liking of the person increased monotonically with each positive trait. This finding and others led to the conclusion that the impression of a person becomes more favorable with each new positive trait. Anderson (1967) referred to this effect as the “set-size effect.” Broadening the scope from impression formation to general persuasion, further research on the effect of amount of persuasive information has generally confirmed the finding made by Stewart (1965) and Anderson (1967). Pelham et al. (1994) refer to the broad positive correlation between amount of persuasive information and persuasion as the numerosity effect. The numerosity effect states that as a default, the more persuasive information a message contains, up to some reasonable limit, the more persuaded people tend to be (see Calder et al., 1974; Norman, 1976; Calder, 1978; Chaiken, 1980; Maddux and Rogers, 1980). Thus, the numerosity effect, whereby presenting more persuasive information leads to more persuasion is quite pervasive (see also Tormala and Petty, 2007). We refer to the inflection-point, after which more information of the same valence no longer causes changes in attitude, as the point of “satiety.” A key question in the research on the optimal number of claims in persuasion is how many claims are needed before informational satiety is achieved.

Recently, the numerosity effect has been related to the elaboration likelihood model (Petty and Cacioppo, 1986a,b) such that participants’ judgments based on the number of considered arguments are observed to differ between high and low elaboration conditions (Tormala et al., 2002). The point of “satiety” might thus differ depending on the cognitive load connected to the attribute information. Elaboration might also impact on the point of “satiety” in that attribute numerosity has been observed to benefit hedonic more than utilitarian options (Sela and Berger, 2012).

An important caveat to the set-size effect or numerosity effect lies in the difference between communication with perceived informational intention, and communication with perceived persuasive intention. There is a crucial difference between the research demonstrating the set size-effect, and marketing studies. In the impression formation literature, the source of the communication, typically referred to as “agent,” has no persuasive intention, only informational intent. The degree to which a target is aware of the agents persuasive intention is referred to as the targets persuasion knowledge (Friestad and Wright, 1994). As consumers have gotten more accustomed to marketers persuasion-intention, they have developed a slightly different way of dealing with information with persuasive intent (Campbell and Kirmani, 2008). Specifically, when dealing with information from a source that has a perceived persuasive intent, subjects will engage in coping-cognitions, in an attempt to maintain a sense of independent and dissuaded view of the product, service or person they are evaluating. The persuasion knowledge model (Friestad and Wright, 1994) is important in this respect, as it changes the focus from message design to message receiver. In doing so, it conveys the notion that the perception of the message is more important than its objective design. The model states that all targets will attempt to hold valid product-, or service-attitudes when faced with a persuasion attempt. In order to maintain a valid attitude toward the product, the target will analyze the persuasion tactics, the effectiveness and the appropriateness of the persuasive agent, and adjust their impression accordingly. Put in terms of CSR marketing, this entails that the perceived social responsibility is more important than the actual or objective social responsibility. Effective CSR communication can be achieved only when the coping efforts of the target is taken into account. It is also important to note that individual differences in skepticism and reactance are likely to induce different levels and styles of coping-cognitions (Clee and Wicklund, 1980; Hong and Page, 1989; Campbell and Kirmani, 2000). Friestad and Wright (1994) call for more research exploring these persuasion dynamics: “An important part of a complete theory of persuasion is, therefore, an explanation of […] aspects of an agents overall behavior that disguise a tactic or that makes its execution seem heavy-handed or transparent to targets.” There are many identified factors in the execution of a persuasion attempt that may make the effort seem heavy-handed or transparent, and thus elicit and increase persuasion knowledge and coping. For instance, prevention-focus or regulation focus in the framing of the message (Kirmani and Zhu, 2007), forced exposure (Edwards et al., 2002), attention-getting tactics (Campbell, 1995), advertising repetition (Kirmani, 1997), and others, have all been identified as factors that increase persuasion knowledge in targets. Increasing the number of claims in a persuasion attempt is another factor that may induce increased persuasion knowledge, as the persuasion-attempt is perceived as more transparent or heavy-handed if the number of claims is perceived as too high (Campbell and Kirmani, 2000; DeCarlo, 2005). It is not all together clear how many claims are optimal for persuasion. Recent research on the optimal number of claims in motivated persuasion has shown that the persuasive effect increases only up to three claims (Shu and Carlson, 2014). Including a fourth claim was shown to increase skepticism and persuasion knowledge rather than persuasion, when consumers know that the message source has a persuasion motive. Through several experiments, Shu and Carlson (2014) demonstrate that three claims produced a more favorable evaluation than two or four claims. They also suggest that coping is the cause of the fall in persuasiveness when a fourth claim is presented, by demonstrating that respondents under high cognitive strain show increased persuasion when being presented with a fourth claim, whereas the non-strained control-group show most favorable evaluation after only three claims. By depleting cognitive resources from the research-subjects, the ability to cope with the persuasive content was reduced.

Part of the reason why over-communication sometimes hampers persuasion may be that when more claims are added, some of the claims are perceived as weaker, or less relevant than the others. Including weak or irrelevant information has been proven to reduce the persuasiveness of a message. Nisbett et al. (1981) refer to this phenomenon as dilution-effect. Dilution-effect is defined as: “A judgment bias in which the presence of non-diagnostic cues, when processed along with diagnostic cues, causes a judge to under-weigh the diagnostic cues” (Waller and Zimbelman, 2003, p. 254). Dilution effects have been documented across many disciplines and research-settings (see Ettenson et al., 1987; Tetlock and Boettger, 1989; Smith et al., 1998; Meyvis and Janiszewski, 2002). Field experiments in economics have documented similar phenomena, referred to as “less is better” or “more is less” effects (Hsee, 1998; List, 2002). In these experiments, bundles of high-quality objects elicit higher willingness to pay than the same bundles, with the addition of some lower quality objects. The use of relatively low quality/low importance claims in conjunction with high quality claims thus appears to be one factor that makes persuasive intent seem more heavy-handed and transparent, which in turn may increase coping, and thus reduce persuasive effect. De Vries et al. (2014) conducted three experiments to explore the role of dilution-effects in communication for and against carbon dioxide capture and storage. They used combinations of highly relevant, moderately relevant and irrelevant claims. Dilution effects were only manifest in positive persuasion, and only when combining highly relevant and irrelevant information. However, interesting, these experiments did not include measures of persuasion knowledge. Experiment 2 in the present research thus represents a partial replication and attempted exploration of the mechanisms behind the findings presented in De Vries et al. (2014).

Summarized, the literature on the number of claims in persuasion suggests four main findings: (a) Increasing the number of claims leads to incremental increase in persuasion, up to a point of satiety. (b) The point of satiety, after which further claims no longer increases persuasion, is reached earlier when the target perceives the agent as having persuasive intent, and later when elaboration and scrutiny is low. (c) Increasing the number of claims can increase the likelihood that the agent is perceived as having persuasive intent. (d) Adding weak claims to bundles of strong claims can dilute the overall persuasiveness of the communication. An important gap in this research is that almost all these findings stem from experiments in positive persuasion, i.e., persuading others to believe that something or someone is good. Whether the same effects would emerge in negative persuasion, i.e., persuading others to believe that something or someone is bad, is largely unknown. This gap is not only of theoretical relevance to psychology. As CSR is becoming an increasingly important part of brand strategy, it is of tantamount importance to understand how consumers perceive different CSR-strategies, how they cope with the persuasive intent of CSR communication, and what evaluative artifacts these coping processes produce (Olsen et al., 2014).

Considering the body of research on good vs. bad perception and judgments (see Baumeister et al., 2001; Rozin and Royzman, 2001), there is good reason to suspect that the dynamics of evaluating negative claims are qualitatively different from those of positive claims. For instance, individuals who spend less time and require less information in order to classify an event, person, or object as bad may have an adaptive advantage. Thus, the consequences of type 2 errors (failing to detect a pattern) in this domain are often more severe than the consequence of type 1 errors (perceiving a pattern where there is none). In literature-studies, the “tragic flaw”, or Hamartia, has been a familiar concept since the Greek dramas. A tragic flaw typically consists of a single failing or transgression that brings about ruin to the otherwise admirable character (Sherman, 1992). Social anthropologist studying purity and contamination in Hindu cultures have noted a negativity bias in that purity is difficult to reach and maintain, while a single act of contagion (like touching a person of lower caste) will instantly contaminate the entire person (Rozin and Royzman, 2001). In social and moral psychology, the negativity effect states that evaluative negative information is weighted more heavily than evaluative positive information in overall evaluations (Kanouse and Hanson, 1971). This effect, sometimes referred to as the positive–negative asymmetry or negativity bias, is considered especially relevant when evaluations entail affective reactions (Lewicka et al., 1992). In judgment and decision-making research, the prospect theory demonstrates that people are more aversive toward small losses than they are positive toward corresponding gains (Kahneman and Tversky, 1979). This effect implies that people tend to be risk-averse over prospects involving gains, while they are risk-seeking when it comes to prospects involving losses (Shefrin and Statman, 1985; Frazzini, 2006). Another consequence of the effect is the tendency of people to sell assets whose price has increased, while keeping assets that have decreased in value. The implication is that people are less willing to recognize losses, but are more willing to recognize gains (Odean, 1998; Weber and Camerer, 1998; Camerer, 2000; Barberis and Xiong, 2009).

Continuing the general ‘bad is stronger than good’ finding into corporate ethics research, Creyer (1997) found that consumers are willing to purchase from unethical companies, but they expect a substantial reduction in prices. The consumers expect ethical corporate behavior as a norm, and are willing to pay a slightly higher price for products from companies who go above and beyond the expected level of ethical behavior. The willingness to pay for products from unethical, normal and ethical companies, respectively, correspond to the curve of prospect theory, in which minor ethical violations are weighted more heavily than corresponding minor ethical advances.

Taken together, the evidence from the ‘bad is stronger than good’-literature, suggests that the number of claims used in a persuasion setting should play a larger role in positive persuasion than negative persuasion. As negative information is processed more thoroughly, the psychological point of satiety should be reached sooner in negative persuasion than positive persuasion. Thus, a positive–negative asymmetry is to be expected, in which the number of claims cause significant changes in the positive domain, whereas the reaction to negative persuasion should be less affected by the numerosity of claims. Borrowing the terms from the Persuasion Knowledge model (Friestad and Wright, 1994), we further theorize that the level persuasion knowledge an individual experiences when being persuaded into believing that something or someone is good, increases as the number of claims increases. As a contrast, the amount of claims used in negative persuasion should not elicit changes in persuasion knowledge, as the point of satiety is reached sooner. There are several reasons to expect that numerosity of claims will fail to elicit differences in persuasion and persuasion knowledge when subjects are dealing with negative claims. Firstly, high coping with negative claims may be evolutionarily maladaptive, as the consequences of adherence and defecting are asymmetrical. As an example, consider an individual in a pre-historic society, being subjected to claims favoring the abolishment of certain foods. Adhering to the advice would take out one of the sources of food from his diet, which is presumably unfortunate but not directly life-threatening. Defecting from the advice may result in much more direct and dire consequences, both in terms of health and social status. Coping when faced with claims saying that something or someone is bad, has presumably been less evolutionarily advantageous than coping when faced with claims saying that something or someone is good. Secondly, modern day consumers are presumably more experienced with positive persuasion, from a lifetime of dealing with marketers (Boush et al., 1994; John, 1999). Negative persuasion is rarely used in marketing, with the notable exceptions of health-behavior ads and political attack ads. Consequently, consumers may activate their persuasion-knowledge and coping schemas more effectively and with greater sensitivity when faced with a positive persuasion attempt, rather than a negative persuasion attempt. Including diluting (weak) claims into sets of strong claims is also theorized to produce asymmetrical dilution-effects across positive and negative persuasion. The subjects being persuaded into believing that a company is good (environmentally friendly), are expected to display a higher readiness to perceive the inclusion of a weak claim as heavy-handed or transparent, alerting them to the persuasion-attempt they are being subjected to. The subjects being persuaded to believe that the company is bad (environmentally aversive), should have a lower readiness to perceive the inclusion of a weaker claim as heavy-handed or transparent, and therefore not utilize the same coping mechanisms.

To summarize, the two related research-questions that stand to be answered in this thesis are; (1) What are the effects of different numbers of claims in positive and negative persuasion? and (2) How does different qualities of claims effect the outcomes in positive and negative persuasion? Based on these research-questions, we state the following four hypotheses:

(1)  The number of claims will have a significant effect on the perception of the company in positive persuasion, but not in negative persuasion.
(2)  The number of claims will have a significant effect on persuasion knowledge and skepticism in positive persuasion, but not in negative persuasion.
(3)  Dilution-effects will emerge in positive persuasion, but not in negative persuasion.
(4)  Dilution-effects on persuasion knowledge will be moderated by dispositional reactance.

## Materials and Pre-Testing

This study was carried out in accordance with the recommendations of the Research Council of Norway, The National Committee for Research Ethics in the Social Sciences and the Humanities, with electronically written informed consent from all subjects. All subjects gave written informed consent in accordance with the Declaration of Helsinki. Before running the experiment, various claims were tested in a population similar to the one used in the experiment. We used CSR-related claims pertaining to an ocean-farming company as setting. There are two main motivations behind this choice. Firstly, CSR persuasion has been studied less extensively than traditional persuasion, even though it is becoming an increasingly important part of branding (Boulstridge and Carrigan, 2000; Sen and Bhattacharya, 2001; Becker-Olsen et al., 2006; Aburdene, 2007; Costa and Menichini, 2013). Secondly, ocean-farming has been both hailed as one of the industries that may potentially be part of the solution to global warming (Asche and Khatun, 2006), as well as criticized for being unsustainable in its current form (Folke and Kautsky, 1992). Our setting thus provides the basis for a plausible and realistic persuasion-attempt, both in favor and against the actions of a company. The participants in the pre-test were exposed to a list of either 10 claims in favor of an ocean-farming corporations’ social responsibility (positive claims), or 10 claims in favor of an ocean-farming corporations’ social irresponsibility (negative claims). Participants were told that all the claims were candidates to be used in a persuasion setting, but that it was up to the participant to rate how strong they felt each claim was. The participants were instructed to allocate 100 points selectively among the claims. Claims perceived as strong were to be awarded more points, and claims perceived as weak were to be awarded few or no points. All 100 points had to be allocated by each participant. The rank order of the presentation of the claims were randomized across trials, to ensure that primacy-, and recency effects did not affect the outcome of the test. A total of 32 student participants rated the positive claims, and 29 student participants rated the negative claims. The four positive and four negative claims with the highest score were chosen to be used in the experiments. **Tables 1** and **2** display these claims (translated to English by the authors), and their mean and median rating.

**Table 1:** Evaluation of positive claims.

Claim	The company has paid to restore 10 km^2^ of destroyed ocean-floor	The company has switched to solar-powered energy	Without reducing the quality in the end-product, the company only uses recycled materials in fodder-production	The company has committed itself to invest 20% of all earnings in technology that will help protect wild salmon and trout
Mean	13,43	17,22	13,25	22,78
Median	12,5	15	14,5	22,5

**Table 2:** Evaluation of negative claims.

Claim	The companys’ activities has destroyed 10 km^2^ of ocean-floor	The company emits over 100 metric tons of CO^2^ each year	The company is unwilling to invest in new facilities. As a consequence, many of the farmed fish escape, increasing the spread of lice, and harming the wild salmon in nearby areas	The farming activities cause the emission of nutrient salts and organic matter, which increase the algae-growth and eutrophication in the inner fjords
Mean	16,37	12,66	17,23	18,17
Median	20	10	15	20

Additionally, two positive and one negative claim were chosen as moderately diluting claims, to be used in the second experiment. These claims were selected because they fulfilled the criteria of having been evaluated as weak but similar in strength, across subjects. **Table 3** displays the diluting claims used in Experiment 2.

**Table 3:** Diluting claims.

Claim	The company has switched to slightly costlier but more eco-friendly lightbulbs in all offices	The company has replaced all its cars with electric cars	The entire company car-fleet consists of high-emission SUV’s
Mean	2,44	4,28	2,77
Median	0,5	1	0

## Procedure and Results

### Experiment 1: The Role of Numerosity

The first experiment was designed to explore the role of different numbers of claims in positive and negative persuasion. Hundred and ninety eight students from a large Norwegian business-school were recruited to the experiment. The participants in the pre-test could not participate in the experiment. Participation was incentivized by lottery of smartphone and serving of pizza. The experiment was done in an auditorium, and subjects used their smartphone, tablet, or pc/mac to read instructions and indicate responses. Web-based experiment-software ensured an even and random distribution of subjects to either positive or negative persuasion. In the positive persuasion setting, participants were exposed to the following vignette (translated by authors):

“Imagine that you are applying for your first job, and that corporate environmental care is of great importance to you. You consider applying at Marine Farming, a large ocean-farming company. A friend of yours already works at the company. In order to convince you that Marine Farming is environmentally friendly, your friend presents the following claims:.”

To ensure validity, the vignette had a 30 s forced exposure setting. The software further randomized these subjects into three subgroups; Group A was exposed to two of the strong positive claims, Group B to three strong positive claims, and Group C to four strong positive claims. The claims were all drawn from the pool of four positive claims presented in **Table 1**. The software ensured that which claims, and the rank order of presentation of claims, was randomized across subjects (except for Group C, in which all four claims were used, and only the rank order of presentation was randomized).

The other half of the sample was randomly allocated to the negative persuasion setting. They were firstly presented with the following vignette:

“Imagine that you are applying for your first job, and that corporate environmental care is of great importance to you. You consider applying at Marine Farming, a large ocean-farming company. A friend of yours works at a competing company. In order to convince you that Marine Farming is environmentally harmful, your friend presents the following claims:.”

This vignette also had a 30 s forced exposure setting. The software further randomized these subjects into three subgroups; Group D was exposed to two of the strong negative claims, Group E to three strong negative claims, and Group F to four strong negative claims. The claims were all drawn from the pool of four negative claims presented in **Table 2**. The software ensured that which claims, and the rank order of presentation of claims, was randomized across subjects (except for Group F, in which all four claims were used, and only the rank order of presentation was randomized). Both vignettes described a source (friend) who spoke of the company in a manner that is congruent with her motives, as we expect that subjects assume that the friend would prefer that the target applies for a job at the same company as the agent.

After having been exposed to both the vignette and the claims, each participant indicated how much they liked the company, how certain they felt about their liking of the company, how believable the claims were perceived, and how relevant they felt the claims were. The participants also gave scores on persuasion knowledge and perceived informational intent by indicating their level of agreement with the statements; “I felt my friend was attempting to influence my choice of employer,” and “I felt my friend wanted to give me information,” respectively. All these outcome-measures were given on 7-point likert-scales. Additionally, each subject completed the 11-item Hong reactance-scale (see Hong and Page, 1989; Hong and Faedda, 1996).

#### Results from Experiment 1

**FIGURE 1:**
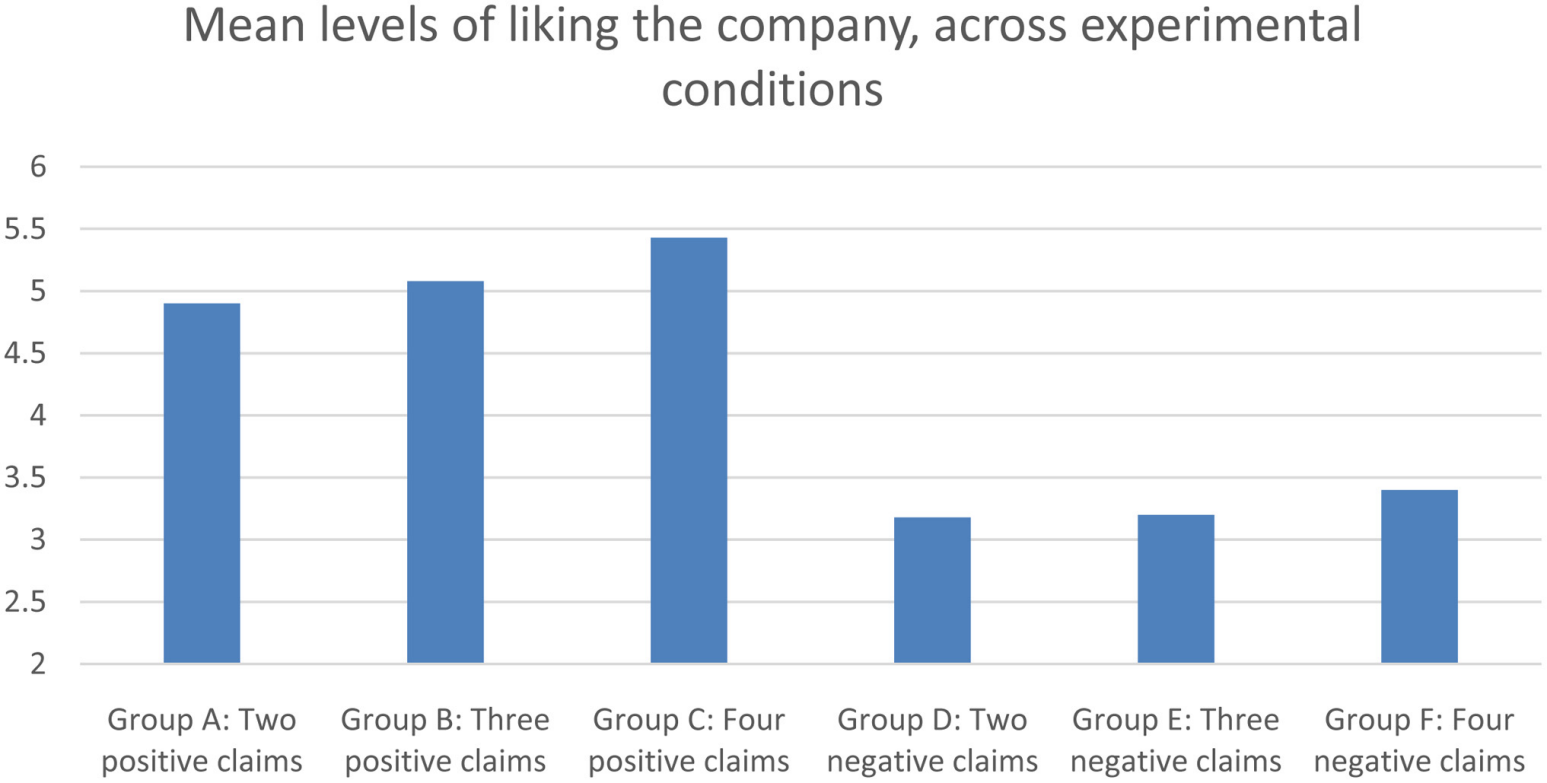
**Single item response of how much the subjects like the company, across experimental conditions**.

Manipulation checks revealed that positive and negative persuasion lead to a significant difference in the perceived likeability of the company, *F*(1,172) = 178,26, *p* < 0.001. There were no overall-effects of amount of claims used. **Figure 1** shows the level of liking of the company among groups who were exposed to either two, three, or four positive claims, or two, three, or four negative claims.

Hypothesis 1 states that there should be significant differences among the different conditions within positive persuasion, not an overall effect of amount of claims. So in order to test Hypothesis 1, we performed planned contrasts between each of the groups in positive persuasion, and each of the groups in negative persuasion, with level of liking the company as the dependent variable. In positive persuasion, univariate analysis of variance revealed significant differences between two and four claims, *F*(1,5) = 7,32, *p* = 0.009, as well as borderline significant differences between three and four claims, *F*(1,2) = 2,97, *p* = 0.09. Surprisingly, there were no significant differences between two and three positive claims. In negative persuasion, liking of company increased marginally with each added negative claim. However, when running the same planned contrasts between different amounts of negative claims, no significant differences in levels of liking the company were found. Based on these findings, we partially confirm Hypothesis 1. It seems that presenting two positive claims “leaves room” for more persuasion, if more claims are added. In negative persuasion, however, a satiety-like finding emerges, in which the level of liking is not affected by the presence or absence of more than two claims.

Previous studies have indicated that behavioral outcomes are seldom predicted by the valence of an attitude alone, but rather by the valence combined with certainty or attitude-strength (Tormala and Petty, 2004). In order to increase our ability to make predictions of behavioral outcomes, subjects in our experiments were asked not only to indicate how much they liked the company, based on the information they had received, but also how certain they felt about that feeling. In order to include both valence and certainty in our analysis, we computed a variable that captures the likeability of the company, multiplied by the certainty-score the subjects gave. Manipulation check of certainty-adjusted liking showed significant differences between positive and negative persuasion *F*(1,3312) = 59,64, *p* < 0.001. There was also a significant main effect of the amount of claims *F*(2,273) = 4,93, *p* = 0.008. Planned contrasts between the different subgroups revealed significant differences between two and four claims in positive persuasion, *F*(1,350) = 4,82, *p* = 0.032. Similar differences were found between three and four positive claims, *F*(1,367) = 6,03, *p* = 0.017. No differences were found amongst the groups in negative persuasion. This finding further supports Hypothesis 1.

**FIGURE 2:**
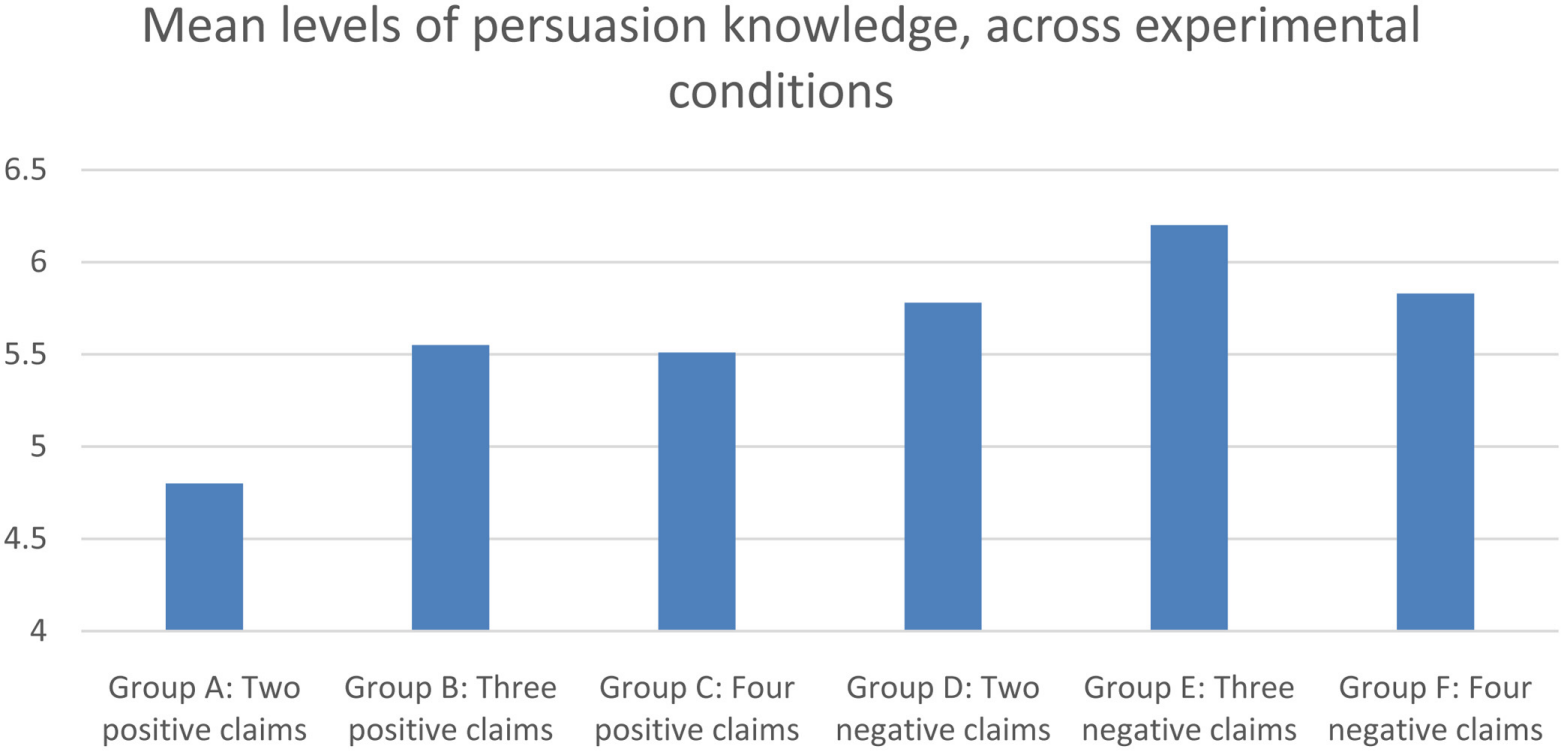
**Single item response of persuasion knowledge, across experimental conditions**.

Using persuasion knowledge as outcome-variable, manipulation checks again revealed significant differences between positive and negative persuasion *F*(1,20) = 13,35, *p* < 0.001. The number of claims also carried a significant main effect *F*(2,5) = 3,69, *p* = 0.027. **Figure 2** shows the level of persuasion knowledge among groups who were exposed to either two, three or four positive claims, or two, three or four negative claims.

**FIGURE 3:**
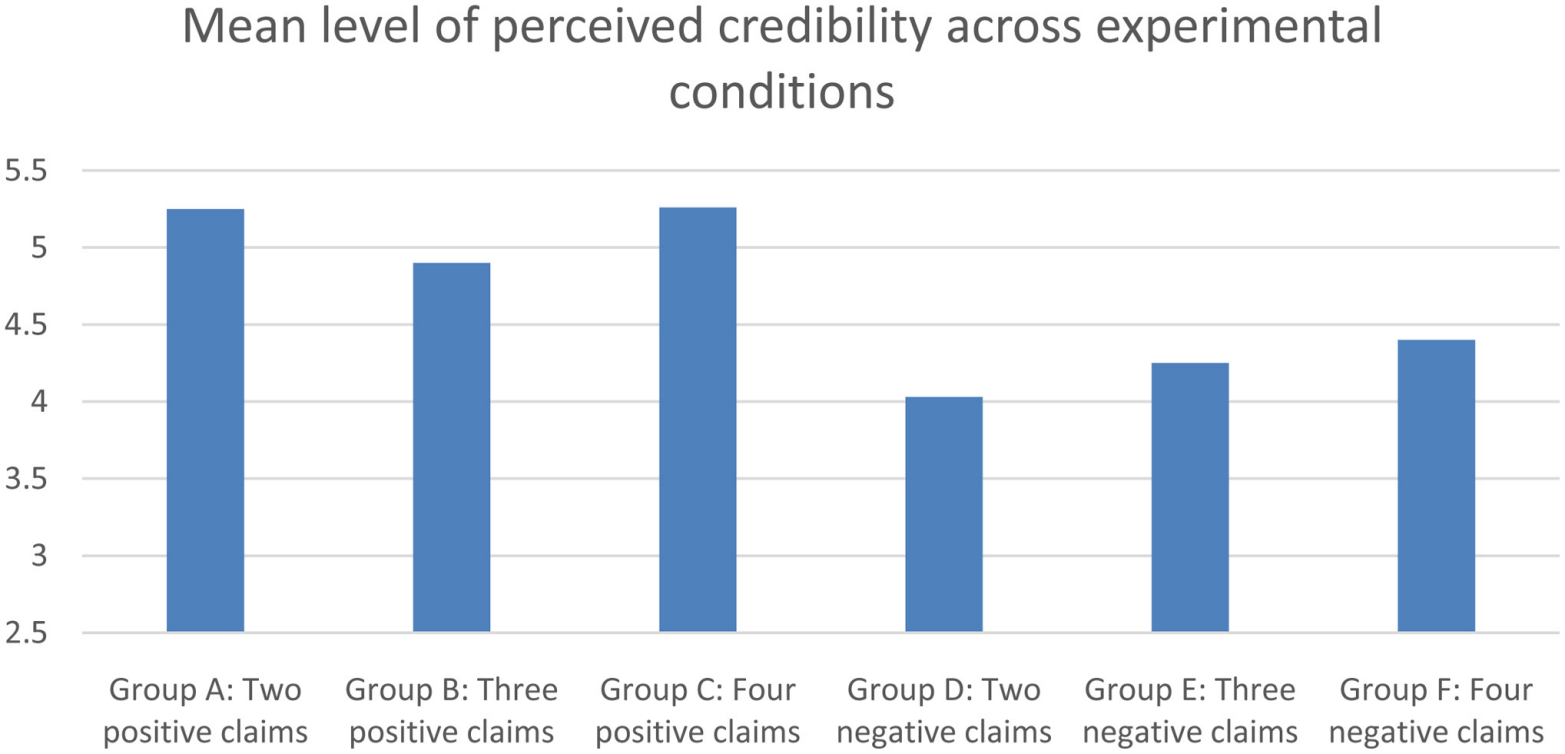
**Single item response of credibility-perception, across experimental conditions**.

An interesting first observation from the analysis is that persuasion-knowledge in negative persuasion is much higher than in positive persuasion. This finding is addressed in larger detail in the discussion part of the article. In order to test Hypothesis 2, we performed planned contrasts between the different groups. The results revealed significant differences in the amount of persuasion knowledge elicited by two and three positive claims *F*(1,9) = 5,71, *p* = 0.020. Going from two to four positive claims also induced a significant shift in the amount of persuasion knowledge elicited *F*(1,9) = 4,44, *p* = 0.039. There was no significant difference between three and four claims. When performing the same planned contrasts for different amounts of negative claims, no significant differences were found. Performing the same analyses with skepticism as outcome variable, we find a significant asymmetry between positive and negative persuasion, in that negative persuasion elicits more skepticism in general *F*(1,42) = 26,31, *p* < 0.001. **Figure 3** displays the mean rating of credibility or believability across groups.

The stark asymmetry in credibility between positive and negative persuasion is also elaborated on in the discussion. Moving on to the planned contrast, we find no significant variance in skepticism with regards to different numbers of claims, in positive or negative persuasion. We further tested whether or not the reported findings could be due to variance in the strength of claims, rather than the numerosity of claims. We computed variables consisting of the sum of strength of claims, based on the pre-test mean and median evaluation of the strength of the claims (see **Table 1**). These variables were used in standard multiple regression models. All the reported significant findings remained, even when controlling for the strength of claims, while none of the reported insignificant findings were explained by strength of claims. Hypothesis 2 thus stand as partially confirmed.

### Experiment 2: Dilution Effects

The purpose of the second experiment was to explore the role of different quality or subjective strength of claims in positive and negative persuasion. Eighty-six students from a large Norwegian business-school was recruited to the experiment. Participation-incentives and practical procedure were identical to Experiment 1. Web-based experiment-software ensured that the participants were randomly distributed to either positive or negative persuasion, in which two of the claims were randomly collected from the list of four claims used in Experiment 1, and one of the claims came from the list of diluting claims presented in **Table 3**. Two different diluters of positive persuasion were used, and one diluter of negative persuasion. The negative diluter used was that the company car-fleet consisted of only high-emission SUV’s. This claim was selected because results from the pre-test indicated that it best fitted the purpose of being a moderately diluting claim. The two positive diluters were chosen on different grounds; the “lightbulb”-claim was chosen because the results from the pre-test indicated that it received a score almost identical to the negative diluter. The “electric cars” claim was chosen because it represents a semantic comparable contrast to the negative diluter. The vignettes and instructions given to the subjects were otherwise identical to Experiment 1.

After exposure to both the vignette and the claims, consisting of one diluting claim and two strong claims, each participant indicated their responses on the same outcome-variables as in Experiment 1. Here as well, each subject completed the Hong reactance-scale (see Hong and Page, 1989; Hong and Faedda, 1996).

#### Results from Experiment 2

Preliminary analysis of the results from Experiment 2 revealed no differences between the two positive diluting claim conditions. To ensure statistical power and symmetry, these groups were combined to a joint diluted positive persuasion group (*N* = 44). All further analysis in this experiment was done with this combined group. No different results were obtained when keeping these groups separate.

To test for dilution-effect, we performed independent samples *t*-tests, where the three strong claims groups were run against their corresponding diluted groups. According to the theoretical predictions, dilution effects were expected to manifest in the positive persuasion domain, but not in the negative domain. However, the findings from the *t*-tests showed that across all outcome variables, in both positive and negative persuasion, no dilution effects were present. This suggests that the presence of a claim with low quality or low subjective strength, in conjunction with two strong claims, has scarce effect on the total persuasion. This finding is similar to the one obtained by De Vries et al. (2014), where only completely irrelevant information produced dilution-effects, and moderately diluting information produced no dilution-effect. Hypothesis 3 is thus rejected.

**FIGURE 4:**
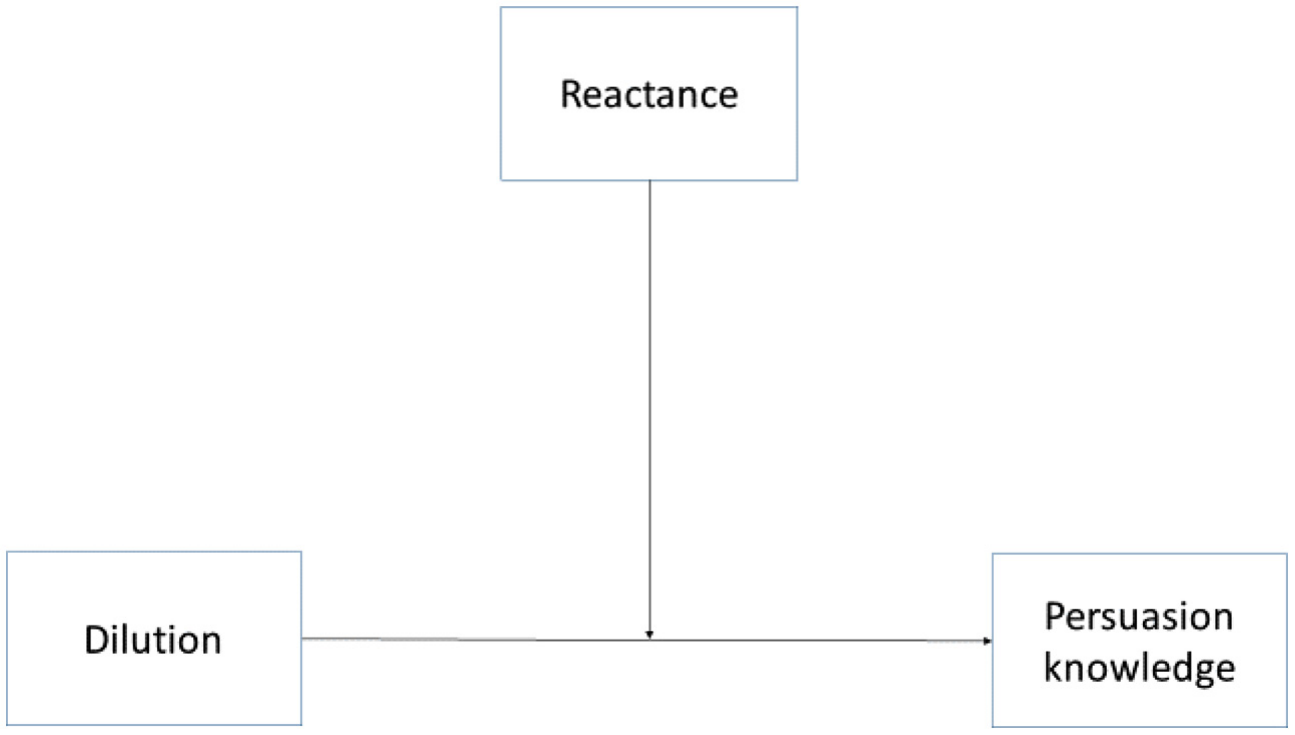
**Moderation model**.

Diluted claims were expected to bring about an increased sense of being subject to a persuasion-attempt, as the total pitch would come across as more heavy-handed. This effect was not manifest in our results, as we found no difference in persuasion knowledge or skepticism between the groups who were exposed to three strong claims compared to the groups exposed to diluted sets of claims. However, both theory and past research on resistance to persuasion suggests that dispositional reactance should give a person a heightened awareness of being subject to a persuasion-attempt. Hypothesis 4 is based on this assertion. In order to test Hypothesis 4, we conducted moderator analyses (see Hayes, 2013), using dilution as independent variable, persuasion-knowledge as outcome variable, and dispositional reactance as moderator-variable. **Figure 4** presents the model described.

The results revealed no interaction when using positive claims. This indicated that different levels of reactance had little or no effect on the amount of persuasion knowledge elicited by including a diluted claim. In negative persuasion, however, a significant interaction was revealed. The effect of dilution of claims, on persuasion knowledge, is dependent upon another predictor, dispositional reactance. **Table 4** shows the values of the model.

**Table 4:** Interaction values.

	Coefficient	*SE*	*t*	*p*	LLCI	ULCI
Constant	13,06	3,80	3,44	0,0011	5,46	20,66
Reactance	–2,04	1,19	–1,72	0,0903	–4,43	0,3313
Dilution	–1,12	0,46	–2,43	0,0179	–2,04	–0,20
Int	0,33	0,15	2,27	0,0264	0,04	0,62

**FIGURE 5:**
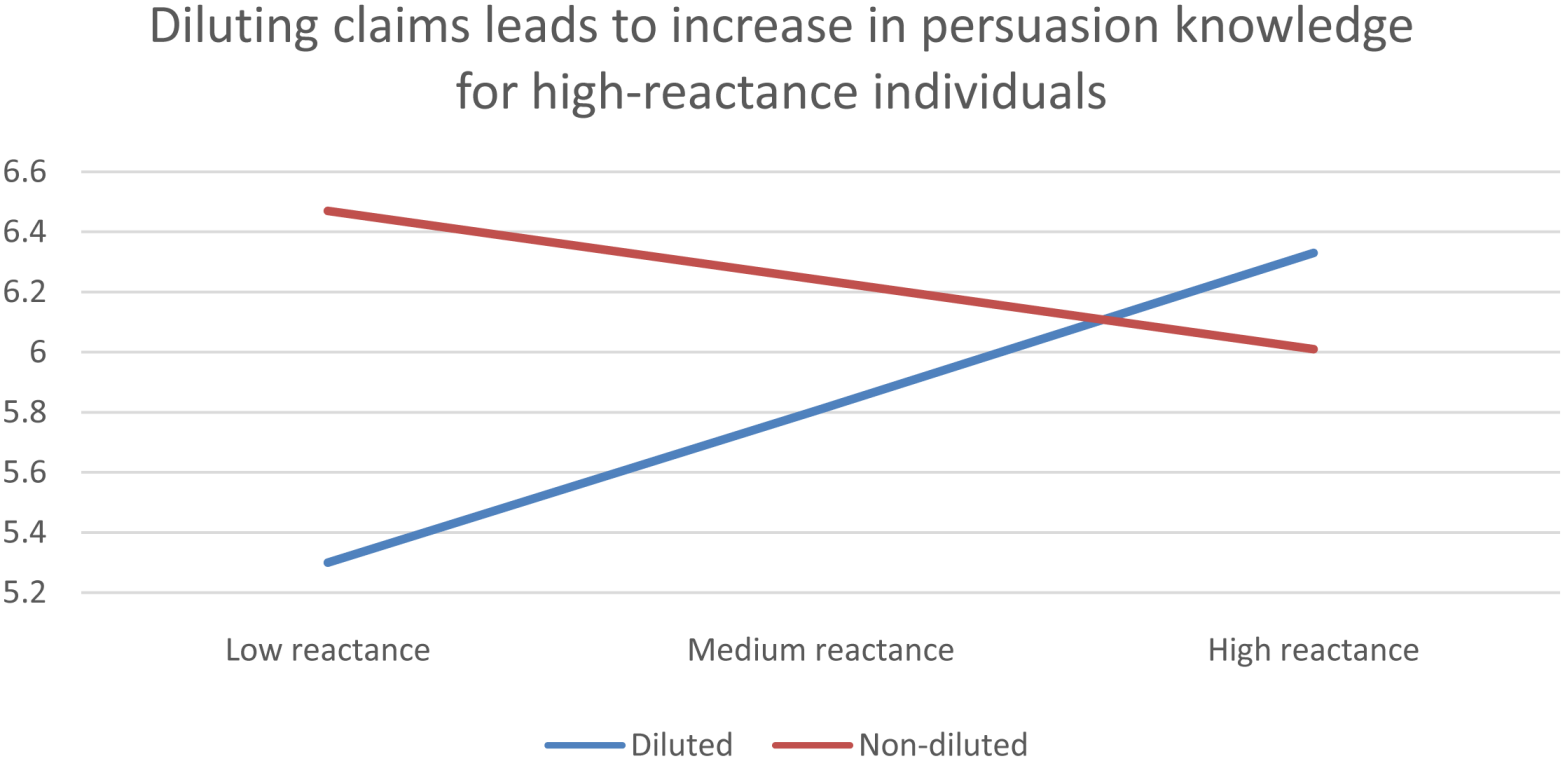
**Interaction**.

Plotting the graphs for level of persuasion knowledge, conditional on dispositional level of reactance, and dilution of claims, it is clear that high-reactance subjects exposed to diluted negative claims showed higher persuasion knowledge, while low-reactance individuals did not. **Figure 5** displays the interaction

## Discussion

### Direct Effects

Our most powerful finding is the large difference in the amount of persuasion-knowledge and skepticism generated by negative claims, relative to positive claims. Our theorizing from the point of evolutionary psychology and consumers past experience with similar persuasion episodes is contradicted by this finding. This is theoretically interesting, as past research on the effects of negative persuasion is limited. We interpret the finding in the light of negativity effect, stating that negative events are given more attention, and weighted more heavily than positive claims. As negative claims receive more attention and elaboration, they also induce an elevated amount of scrutiny in the targets interpretation of the claims. The target who gives more attention and performs a deeper elaboration of the claims is more likely to make attributional inferences into the motivation of the agent, resulting in an increase in their awareness that they are being subjected to a persuasion-attempt. Put differently, they increase their persuasion knowledge. The heightened level of scrutiny also induces a higher level of skepticism, making the target perceive the claims as less believable. The high levels of persuasion knowledge and skepticism revealed in the negative persuasion groups correspond well with the limited effect negative persuasion had on overall likeability of the company. The positive persuasion induced a mean liking that was higher than the one for negative persuasion. Given that the neutral point on this outcome variable is four, the results clearly indicate that the impact on likeability generated by positive persuasion was far greater than that of negative persuasion. The reason for this difference seems to lie in the increased coping in negative persuasion relative to positive persuasion. However, the limitations of the current study leaves room for alternative, or supplemental interpretations. In the current study, the target was presented as “considering applying at Marine Farming.” This may be perceived as stating that the target already held a positive view of the company, and a behavioral intention that leaned more toward applying than not applying. Subjects may have interpreted the vignettes as saying that the positive persuasion agent tried to enhance or affirm a behavioral intention that was present to begin with, while the negative persuasion agent tried to stop and alter a behavioral intention. As such, it may be the case that arguing against the application is perceived as a more invasive or heavy-handed action than arguing for it. However, as both vignettes described the target as “considering applying,” another interpretation may be that the target already has a slightly negative view of the company, hence the need for consideration. In line with this interpretation, negative claims would be conceived of as more affirming of an attitude that is already present, while positive claims are perceived as a more invasive attempt at attitude-change. We therefore disregard this potential interpretation. Another confounding element in the present study may be that the person arguing for the company is currently working there, while the person arguing against the company works at a competing firm. The different positions of the sources may be considered a confounding variable, which might explain the heightened skepticism and persuasion knowledge among the subjects exposed to negative persuasion. The vignettes were designed in this way to secure a sense of motivation on behalf of the source in both the negative and positive persuasion setting. Future experiments should attempt to remove this confounding variable, while maintaining realism in the congruency of source and motivation. The present study described a typical scenario for positive and negative persuasion, in which a representative of a company speaks well of her employer, or ill of a competing company. We consider the opposite scenario, in which someone speaks well of the competition, and ill of one’s own company, as less realistic. Indeed, De Vries et al. (2014), attempted to use claims that were incongruent with the motives the organization in question is assumed to act upon (an industrial organization that spoke well of CO^2^ capture and storage). They found that this breach of realism in the design of stimuli made the subjects confused, to the extent that their responses failed to pass the manipulation check. In our experiment, the source of the message is positioned in a way that allows for congruency between her motivations and the content of her claims. On this basis, we can conclude that positive claims made from someone within a company generates far less skepticism and persuasion knowledge than negative claims made from someone at a competing company. Although we are unable to pinpoint whether this effect stems primarily from negativity bias or the relative position of the source of the claims, the finding is nevertheless theoretically and pragmatically interesting, as it arose from a realistic description of events.

Our secondary sets of findings show that the effects of different amounts of claims in persuasion are asymmetrical across the positive–negative divide. This finding resonates well with the theoretical predictions from negativity effect and numerosity effect. More specifically, we demonstrate that the amount of claims induce quite small variances in outcomes overall, but the significant changes are all within positive persuasion. This finding also corresponds well to the predictions made by past work on positive–negative asymmetry. We assert that receiving more and more information about how environmentally responsible or irresponsible a company is, will affect ones perception of the company, up to a certain point. Past this point, more information of the same valence will no longer produce changes in the impression of the company. We refer to this theoretical inflection point as a satiety point, as there is no longer any effect of additional information of the same valence. The finding that more information elicits changes in the effect of positive persuasion, but not in negative persuasion, is interpreted in the light of this satiety-model of persuasion. Our finding demonstrates that the psychological level of satiety is reached sooner in negative persuasion than in positive persuasion. The reason for this is that negative claims are weighted more heavily, given more attention, and elaborated on more thoroughly, than positive claims. Consequently, less information is needed before the point of satiety is reached. This finding resonates well with prospect-theory (Kahneman and Tversky, 1979), in that the slope on the graph is steeper in the area of negative information (losses) than in the area of positive information (gains). It also corresponds well with the notion of contagion, put forth by Rozin and Royzman (2001), in that perceived virtue stems from many, consistently positive behaviors, while a single act of immorality elicits immediate contagion, and further acts of immorality are superfluous in changing the impression for the worse. Here as well, it is possible to argue that the position of the source plays a confounding role in the interpretation of the claims. However, even though the claims presented in negative persuasion are perceived as less believable than the ones presented in positive persuasion, they are still perceived as more believable than neutral (mean believability rating >4). Another possible confounding aspect to our design is the fact that the claims used are semantically different, and were evaluated differently in the pre-test. However, the variance in strength within the four positive and four negative claims is very similar. The difference in median score from the weakest to most powerful claim is 10 points, in both sets of claims. And as the list of two, three, or four claims were randomly generated for each research-subject, we believe that it is unlikely, but not impossible, that the results could be caused by differences in the strength of the claims. Within positive persuasion, our findings show support for the numerosity effect (see Pelham et al., 1994). Our findings contradict the “charm of three” finding, demonstrated by Shu and Carlson (2014), in which three claims consistently generated more liking and less persuasion knowledge than four or two claims. Instead, we find that four claims significantly outperforms two and three claims, even though persuasion knowledge is increased. One of the differences between our design and that of Shu and Carlson may be that we assess persuasion in a CSR setting, rather than in a traditional marketing setting. The claims we therefore use are more specific in nature than the claims used by Shu and Carlson (2014).

### Interaction Effects

Finally, we demonstrate that including moderately diluting claims in bundles of claims give no direct effect on the outcomes of persuasion. This finding is consistent with previous research on moderately diluting claims (Tetlock and Boettger, 1989; De Vries et al., 2014). The implication of this finding is that the subjective quality of the claims used in CSR communication has less effect on the outcome than one would intuitively imagine. We interpret this finding as being fairly consistent with the phenomenon of scope insensitivity or scope neglect (see Desvousges et al., 1992). This effect states that, in lieu of available reference-points, different levels of positive and negative impact on the wellbeing of people and ecosystems are unconducive to persuasion. This effect has been studied experimentally in philanthropy and environmentalism. For instance, when asked how much they would be willing to pay to save 2000, 20,000, or 200,000 migratory birds from uncovered oilponds, the respondents average answers were 80, 78, and 88$, respectively. Similar experiments showed that residents would pay little more to clean up all polluted lakes in Ontario than polluted lakes in a particular region of Ontario (Kahneman, 2004). Furthermore, residents of four western US states would pay only 28% more to protect all 57 wilderness areas in those states than to protect a single area (McFadden and Leonard, 1993). One proposed explanation for scope neglect is the “valuation by prototype”-hypothesis, suggesting that the mental representation of the different options usually consists of single, representative and emotionally charged exemplars (such as a single bird drenched in oil), rather than numerical variables (Kahneman et al., 2000). The results from our experiment are compatible with scope neglect, as the subjects responses indicate that the attitude toward the company is unaffected by the scope of the claims, and more affected by the prototypical direction those claims point (good/bad). Only when adding dispositional reactance as another predictor variable were we able to identify a significant effect of dilution. Based on available theory, it is easy to understand why the level of dispositional reactance is associated with the amount of persuasion knowledge elicited by diluted claims. However, it is difficult to interpret, based on available theory, why this phenomenon is asymmetrical across the positive–negative divide, and only manifest within negative persuasion. Hence, we report it here as a singular finding, and leave the interpretation for future research.

### Contribution, Implication, and Future Research

The present study is one of the few studies to investigate persuasion in both positive and negative directions. Recently, Rozin and Royzman (2001) found that negative information was more powerful than parallel positive information. Based on this and other findings, loss-framed appeals have been launched as more persuasive than gain-framed appeals (e.g., Johnson et al., 2005). However, negative frames have proven to be more persuasive than positive ones predominantly when participants’ processing of the information is in-depth (Block and Keller, 1995). The impact of negative message framing is thus dependent on degree of elaboration, it seems (Jones et al., 2003). Nevertheless, negative framing not only requires deep processing in order to have a greater impact than positive framing, it also stimulates more effortful and thorough information processing in itself (Kuvaas and Selart, 2004).

The present study adds to this literature by assessing in particular how persuasion knowledge and skepticism are elicited by positive vs negative persuasion. Our main finding, that morally motivated positive framing of one’s employers’ green activities generates far less skepticism and persuasion knowledge than negative framing of competing firms harmful activities, is of both theoretical and managerial value. The value of the finding comes largely from the fact that the design of the study is realistic and motivationally congruent. From a managerial point of view, the results of this study imply that word-of-mouth accusations on behalf of a competitor are largely inadvisable, as they not only face the risk of being condemned as inappropriate, but that the entire persuasion-attempt risks coming across as heavy-handed or transparent, as well as less believable. Highlighting one’s own laudable efforts seems to be a better persuasion tactic. From a theoretical point of view, our results indicate that there seems to be a positive–negative asymmetry in persuasion, not only concerning the consequences of the persuasion *per se*, but also the consequences using different numbers of claims. This deepens our understanding of the interaction between the numerosity effect and the negativity bias. However, as with most persuasion-experiments, we cannot assert with certainty that these results can be generalized to any persuasion setting. We recommend that future research should attempt to isolate the mechanisms behind the positive–negative asymmetry documented here, while maintaining ecological realism and congruency in the design of stimuli.

## Conflict of Interest Statement

The authors declare that the research was conducted in the absence of any commercial or financial relationships that could be construed as a potential conflict of interest.
